# Expression Profiling of Microarray Gene Signatures in Acute and Chronic Myeloid Leukaemia in Human Bone Marrow

**Published:** 2015-03-15

**Authors:** E Sakhinia, MA Estiar, S Andalib, A Rezamand

**Affiliations:** 1 Connective Tissue Disease Research Center, Department of Medical Genetics, Tabriz University of Medical Sciences, Tabriz, Iran.; 2 Department of Medical Genetics, School of Medicine, Tehran University of Medical Sciences, Tehran, Iran.; 3 Neurosciences Research Center, Imam Reza Hospital, Tabriz University of Medical Sciences, Tabriz, Iran.; 4 Department of Pediatrics, Children Hospital, Tabriz University of Medical Sciences, Tabriz, Iran.

**Keywords:** Microarray, PolyA PCR, RT-PCR, Gene Signature, Myeloid Leukaemia

## Abstract

**Background:**

Classification of cancer subtypes by means of microarray signatures is becoming increasingly difficult to ignore as a potential to transform pathological diagnosis; nonetheless, measurement of Indicator genes in routine practice appears to be arduous. In a preceding published study, we utilized real-time PCR measurement of Indicator genes in acute lymphoid leukaemia (ALL) and acute myeloid leukaemia (AML) as a way of application of microarray gene signatures. More to the point, the specificity of such genes for this distinction was investigated by their measurement in cases afflicted with chronic myeloid leukaemia (CML) and with normal bone marrow (BM).

**Material and Method:**

Mononuclear cells were sorted into unselected (total), CD34+ve, and CD34-ve fractions, mRNA globally amplified by using PolyA PCR. Moreover, the level of expression of 17 Indicator genes was identified by using real-time PCR.

**Results:**

No statistically significant difference was observed in expression for any gene among CML cases. Cyclin D3 (p≤0.04) was exclusively upregulated in CML in the CD34+ fraction, notwithstanding upregulation of HkrT-1 (p≤0.02) and fumarylacetoacetate (p≤0.03) in AML. HOXA9 experienced a non-significant upregulation in AML; however, in combination with proteoglycan 1 distinguished between AML and normal samples in the CD34- fraction in unsupervised clustering. Unsupervised clustering distinguished among AML and the other diagnostic groups.

**Conclusion:**

The evidence from the present study suggests that the genes discriminatory between ALL and AML are uninformative in the context of CML and normal BM, excepting for distinction with AML.

## Introduction

Presently, diagnosis as well as monitoring of the acute myeloid leukaemia (AML) and chronic myeloid leukaemia (CML) is commonly reached at the level of cell morphology, protein expression, and cytogenetics(1). Recent attempts have further stratified the disease by expression of gene signatures, namely, Indicator genes, which can be assessed by microarray profiling (2). Such attempts offer more specific diagnosis, prognostication, and the development of tailored treatment (3). Nevertheless, an applied way is now essential to evaluate such gene sets in routine clinical practice.

Application of cDNA arrays is restricted because of its cost and its need to fairly large amounts of RNA in routine clinical practise. In order to surmount these obstacles, a global amplification approach, which is known as polyA PCR, was used in the current study. PolyA PCR co-ordinately amplifies cDNA copies of all polyadenylated mRNAs and creates a PCR product (polyA cDNA), whose composition reflects the relative abundance of all encoded genes in the starting sample (4). This can be used for very small samples, incorporating single cells (4). Real-time PCR measurement through using gene specific primers and probes of the expression levels of specific Indicator genes leads to gene signatures detection within the polyA cDNA and enables expression profiling of very low amounts of starting material (5). We investigated this method application through measurement of levels of gene expression in in 17 indicator genes of bone marrow (BM) of individuals suffer from AML chosen from a preceding microarray investigation by Golub et al. (6). Most 17 of the genes showed an expression in AML and ALL similar to that reported by Golub et al. (6), showing diagnostic utility of the method (7). Such Indicator genes were chosen from a microarray comparison of expression profiles in AML and ALL. However, whether the Indicator genes found in this comparison are specific to this diagnostic scenario or they can be applied for assessment of other myeloid disorders, for instance chronic myeloid leukaemia (CML). It is of crucial importance to establish the genes’ specificity for usage of microarray gene signatures. In fact, the high specificity dictates the signatures to be exclusively informative in narrowly defined diagnostic scenarios. Great care will be essential in choosing suitable panels of genes for diagnostic assessment if as part of the previous work, BM samples were obtained from cases with CML and morphologically normal BM. In the present study, the expression profile of the 17 Indicator genes applied to distinguish AML from ALL were studied in these opportunistically obtained samples of CML and normal BM in order to find whether the gene signature for distinction of AML and ALL can be used in other myeloid malignancies.

## Materials and methods

**Sample acquisition**

BM aspirates were obtained from 26 subjects with AML, 18 subjects with CML, and 12 subjects with morphologically normal BM. Clinical characteristics of the subjects are tabulated in Table 1. All BM aspirates were provided into Hanks buffered saline solution (HBSS) with 100 units of preservative free heparin and 1% penicillin, streptomycin, and amphotericin (PSA). Each BM aspirate sample was centrifuged to eliminate supernatant and fat. The cell pellets were undergone density gradient centrifugation over Histopaque for 35 minutes at 400g, in order to obtain mononuclear cells from the interface phase. Afterwards, the mononuclear cells were washed in HBSS, re-pelleted, and re-suspended in 600μl of MACS buffer (PBS pH 7.2 supplemented with 0.5% BSA and 2mM EDTA). One fraction (200μl) of the cells was eliminated and stored at 4ºC as the total BM aspirate cell fraction (TBM). Magnetically activated cell sorting was carried out for the remaining 400μl in order to generate CD34 positive and CD34 negative cell fractions. 

**CD34 Cell Sorting**

Fc-receptor mediated binding of CD34 Micro Beads to non-target cells was blocked through adding 25µl of FcR Blocking Reagent, followed by incubation with 25µl of CD34+ve microbeads for 30 minutes at 4ºC. Subsequently, the cells were washed; 400µl of MACS buffer was added; the cells centrifuged at 20ºC for 10 minutes at 300g and the cell pellet was re-suspended in 500µl of MACS buffer. This process was followed by loading of the cells suspension onto a prepared MS column in a Mini MACS magnetic separator. Afterwards, the column was rinsed twice with 500µl of MACS buffer to obtain the CD34-ve fraction. The column was then eliminated from the magnetic separator and 1000µl of MACS buffer was also added to flush out the CD34+ve fraction. 

**Global amplification of Poly Adenylated mRNAs (PolyA RT-PCR)**

As recommended by the manufacturer, total RNA was extracted from all the fractionated samples (total, CD34+ve, CD34-ve) by means of an RNeasyTM mini kit (Qiagen). Global amplification of cDNA samples of all encoded genes (polyA PCR) was undertaken as previously reported (4, 5). Briefly, 0.5 microgram total of messenger RNA, which was suspended in1 μl of buffer, for each sample was added to 10 μl of fresh 1st Lyse solution (50mM Tris-HCL PH 8.3, 3mM MgCl2, 75mM KCl, 0.5% Nonidet P-40, 10 μM dNTPs, 23 nM dT24 oligo ( 5`- CAT CTC GAG CGG CCG (T)24 -3`), and 0.2 μl RNase inhibitor) and subjected to the following thermal profile: 65^o^C for 1 minute, 25^o^C for 3 minutes, and 4^o^C for 15 minutes. Having addition of AMV reverse transcriptase (0.5µl), it was incubated at 37^o^C for 15 minutes, after which reverse transcriptase was denatured at 65oC for 15 minutes. The cDNA strands were tailed through adding an equal volume (11.5μl) of tailing buffer to a final concentration of 200mM potassium cacodylate, 25mM Tris-HCl, pH 6.6 at 25^o^C, BSA 0.25 mg/ml & 1mM dATP, followed by addition of 0.5μl (25 units) of terminal deoxynucleotidyl transferase (tdt) and incubation at 37^o^C for 15 minutes. Tdt was then denatured at 65^o^C for 15 minutes. Thereafter, 7.5μl of this tailed cDNA was utilized for subsequent polyA PCR and the remainder stored at -20^o^C. 7.5μl tailed cDNA was added to a thin walled 0.2ml PCR tube including a PCR mix at a final concentration of 10mM Tris-HCl, pH 8.3, 50mM KCl, 2mM MgCl, 100μg/ml BSA, 0.05% Triton X-100, 2mM dNTPs, and 12μM NOT I-T24 primer. 1.5μl Ex-Taq DNA polymerase (TaKaRa) was then added and PCR was carried out by using the following thermal protocol: 94^o^C 3 minutes, 25 cycles of 94^o^C 1 minute, 42^o^C 2 minutes, 72^o^C 6 minutes, which followed by 25 cycles of 94^o^C 1 minute, 42^o^C 1 minute, and 72^o^C 2 minutes. Subsequently, polyA cDNA products were adjusted to a final concentration of 0.5 microgram/ml and stored at –20^o^C.

**Preparation of human genomic DNA **standards as gene specific quantity marker 

A dilution series of human genomic standards for calibration of real-time PCR was produced by using human genomic DNA (hgDNA). hgDNA (Promega) was also homogenised by sonication and serially diluted in TE buffer to apply standards wherein the number of DNA molecules ranged from 1.5X104 to 24/μl (8). Each standard was thereafter aliquoted into 1ml amounts and stored at –20 ^o^C.

**TaqmanTM real-time quantitative PCR**

All in all, 50 Indicator genes discriminatory between AML and ALL were shown by Golub et al. (6), (1999). Reassessment of the same data made by Thomas et al, (2001) (9) presented a further group of genes which distinguish among AML, ALL. Seventeen Indicator genes were selected from this gene signature for evaluation in the present investigation involving AML – cystatin c, leptin, fumarylacetoacetate, CD33, HOXA9, adipsin, proteoglycan 1, LTC4 synthase, Lyn, ALL – c-myb, MB-1, cyclin D3, hSNF2, RbAp48, proteasome iota, HkrT-1, E2A. These genes were evaluated in patients with AML and ALL for a larger investigation (7). As a part of the study, CML patients were collected to compare the expression of AML/ALL Indicator genes in a separate but related case group. For each gene, TaqmanTM PCR primers and probes were designed by means of Primer Express Software (Perkin Elmer/Applied Biosystems) shown in Table 2. As recommended by the manufacturer, for each gene, TaqmanTM PCR was performed to 1ng polyA cDNA from each sample and to 10 microlitre of all serial human genomic standards, by using a TaqmanTM Gold kit. In addition, as recommended by the manufacturer, samples were assessed by using an ABI Prism 7700 sequence detection system (PE Applied Biosystems). 

The expression levels of three housekeeping genes, that is to say, IF2-beta, GAPDH, and human ribosomal protein S9 mRNA were evaluated by means of RT-PCR in each sample. Copy numbers obtained for the mean (Mhouse) of the three housekeeping genes (IF2-b, GAPDH and RbS9) in each sample were divided by the highest Mhouse in all samples giving rise to a normalization correction factor. Subsequent to real time PCR amplification and quantification of the selected genes, this factor was applied for normalization of expression levels of each of the 17 measured genes.

**Statistical analysis**

Initial statistical assessment revealed that the data were not normally distributed so non-parametric analyses were applied. Statistical assessment of the expression levels of the 17 genes specifically was carried out in each diagnostic group by using the following non-parametric tests: test of median, the U of Mann Whitney, and Kruskal-Wallis. P≤0.05 was considered as statistically significant. In order to assess the relationship among all the studies genes, non-parametric spearman's rank correlation test was applied. More to the point, these calculations were done separately for each individual fraction (total, CD34+ve, and CD34-ve). The ranking of all the expression levels of Indicator genes among different groups was analyzed by means of the Kurskal-Wallis (K-W) test and presented as the mean rank statistical difference. All the tests were conducted by using the statistical package for the Social Sciences (SPSS) software (release 11.0 SPSS Inc., Chicago, IL, and USA). Because the expression levels for all the genes fell across a large range, the natural logarithm (LN) was applied to plot all values. In fact, LN allows a wide range of values for being visually compared (ln x = logarithm of x with the base e, where e is an irrational number with the approximate value of 2 72).

**Cluster analysis**

Unsupervised cluster analysis of the normalized gene expression values for each sample was conducted by using Cluster and TreeView presented by Eisen laboratory (http://rana.lbl.gov/EisenSoftware.htm).

## Results

**Global amplification and Real-time PCR**

measurement of Indicator genes PolyA cDNA was produced from mRNA, which was extracted from each of the fractions in each sample. After normalization to the copy numbers obtained for the mean (Mhouse) of the three housekeeping genes, the copy number of each gene was identified by reference of the real-time PCR expression level to serial standards of human genomic DNA. In order to indicate reproducibility of the method, the copy number was also identified in duplicate tests for each sample.

**Comparison of three BM fractions**

The expression of each housekeeping gene was assessed and compared in the 3 fractions namely, total, CD34+ve, and CD34-ve. No significant difference was seen in the housekeeping genes’ expression among the fractions or the different diagnostic groups.

The findings of statistical comparison of the level of gene expression for each Indicator gene in the different diagnostic groups are shown in details below. All figures, the natural logarithmic values of each gene in the different diagnostic groups are shown for all genes indicating a significant difference among groups for each comparison. The rank means for all genes (significant and non-significant) among each group are also illustrated.

**AML versus CML**

**Figure 1:**
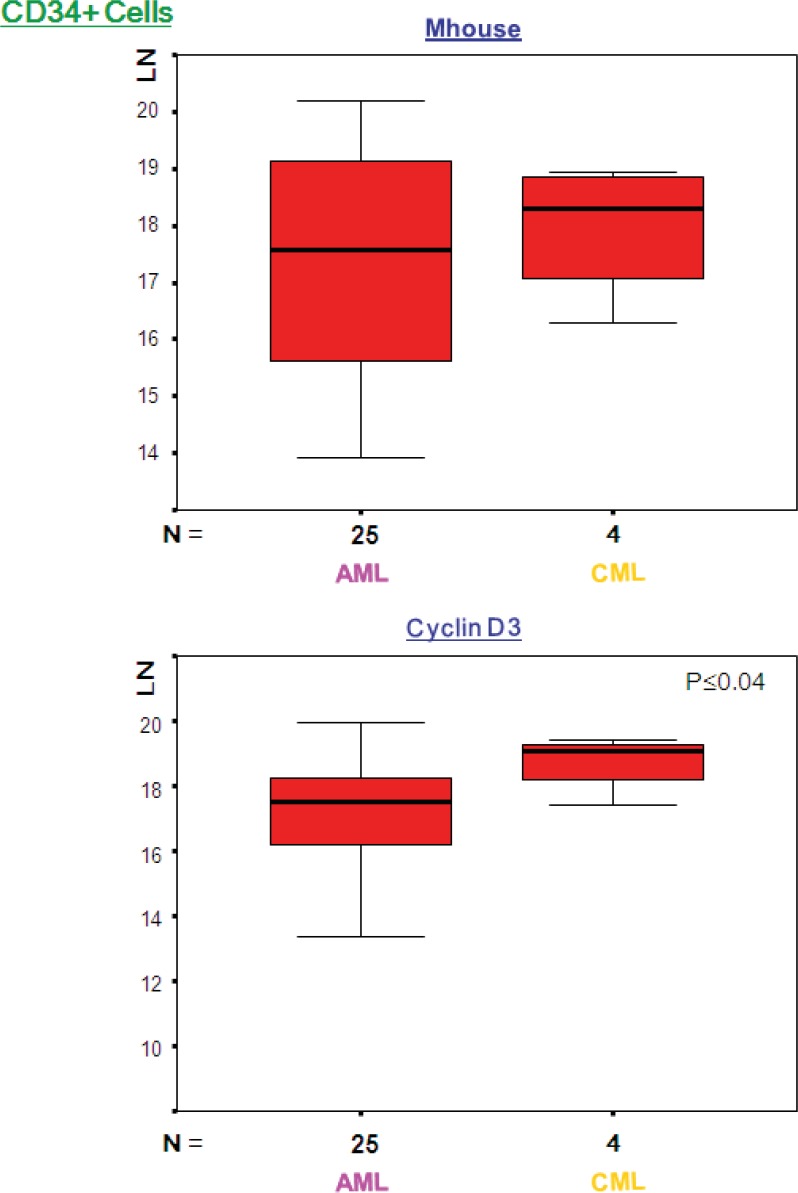
Expression levels (LN) of genes with statistically significant difference between AML and CML. The above box plots show significant difference in expression for cyclin D3 in the CD34 positive fraction. There was no significant difference in expression level of Mhouse between AML and CML. LN, natural logarithm; Mhouse, mean of three housekeeping genes expression levels; N, number of samples in each group.

**Figure 2:**
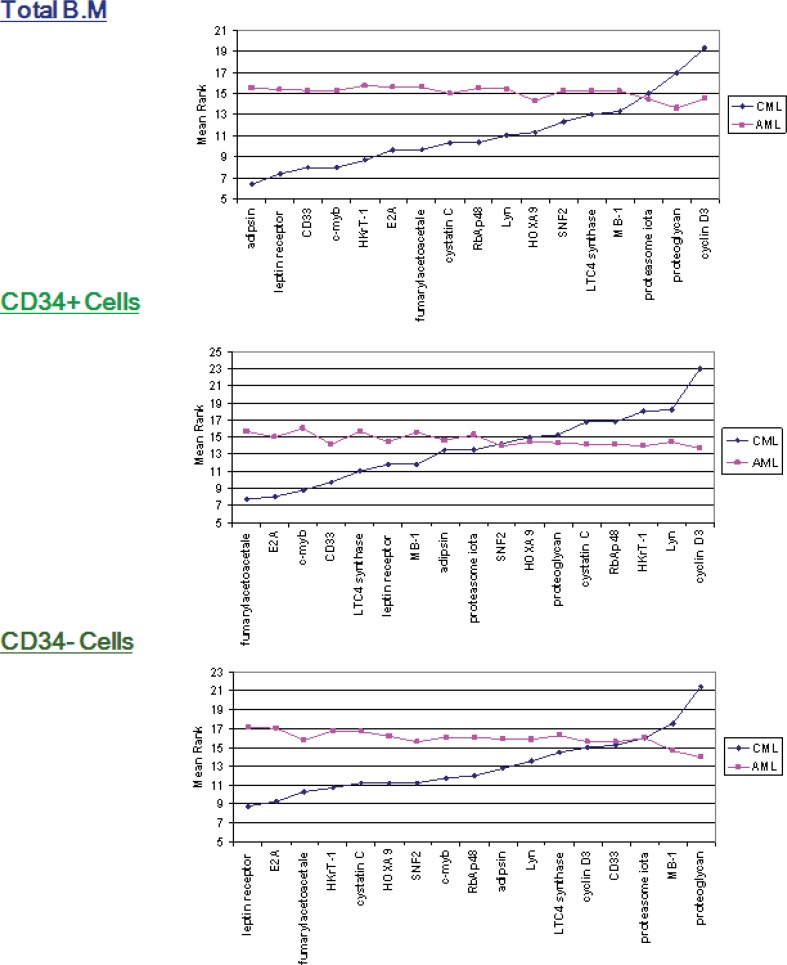
Mean ranks for Indicator genes in CML and AML in each of the three fractions. Mean ranks (y-axes) for each gene, calculated using the Mann–Whitney in the CML and AML groups (total, CD34+ and CD34-), sorted based on gene mean rank for CML

Cyclin D3 was expressed at significantly higher levels in CML (p≤ 0.04); however, the remainder of the genes indicated that there was no significant difference between the two groups (Figs 1 and 2). The mean ranks for each of the genes between AML and CML indicated upregulation of cyclin D3 in CML in comparison with AML in the CD34+ve fraction, whereas it was upregulated in the total BM fraction, and there was no significant difference (p≤0.39). The mean rank analysis revealed downregulation of adipsin in CML in the total BM fraction, although there was no significant difference (p≤0.08). The mean rank plots showed general trends in the relative expression levels for each Indicator genes between AML and CML. In the total BM and CD34-ve fractions, most of the genes were highly expressed in AML, compared to CML; however, in the CD34+ fraction similar numbers of genes demonstrated a higher expression in AML and CML.

**AML versus Normal BM**

Statistically significant different expression was found in several genes between AML and the normal BM. In particular, there was an upregulation in the total BM fraction, adipsin (p≤0.03), and HkrT-1 (p≤0.02) in AML. Nevertheless, an upregulation was observed in fumarylacetoacetate (p≤0.014), and LTC4 synthase (p≤0.014), HOXA9 (p≤0.01), and c-myb (p≤0.01) in the CD34 positive and negative fractions, in AML, in comparison with normal BM (Fig 4). There is a difference in expression between AML and normal BM in the mean rank plot for each gene (Fig 5), whereas a wide separation of the curves for AML and normal BM was observed.

**AML versus CML and Normal BM**

A significant difference was found in expression for HKrT-1 (p≤0.02) and fumarylacetoacetate (p≤0.03), both of which were upregulated in AML, as compared to the other diagnostic groups in the total bone marrow and CD34+ve fractions. Adipsin was demonstrated a non-significant (p≤0.08) lower expression in CML, in comparison with the other groups in the total BM fraction; however, cyclin D3 experienced a higher expression (p≤0.08) in CML in the CD34+ve fraction. Furthermore, HOXA9 (p≤0.06) and c-myb (p≤0.08) experienced a non-significant upregulation in AML, in comparison with the other groups in the CD34-ve fraction., A similar pattern of expression for the Indicator genes in the normal and CML samples, especially in the total BM fraction was found in the mean rank analysis for the comparison of AML, CML, and normal BM. However, there was a similar expression in the CD34+ and –ve fractions in these two groups (Figs 6 and 7).

**Cluster analysis**

Unsupervised clustering was carried out by using the normalized data from each fraction (Fig 8). In each fraction, most normal BM samples clustered together. In all three fractions, however, the housekeeping genes clustered together (Figs 8a-c). Most CML samples were shown to cluster together in the total BM (Fig 8a) and CD34-ve fractions (Fig 8c). Clustering of the AML and normal samples alone was conducted in the space of the genes upregulated in AML (from Golub et al, 1999), in the total BM fraction, wherein all the normal BM samples clustered together (Fig 8d). In order to highlight the results, the distinction between AML and normal samples could also be further drawn by clustering in the space of proteoglycan 1 and HOXA9 alone in the CD34-ve fraction (Fig 8e). Such genes were chosen for clustering from the rank mean plot of their expression in AML, CML, and normal BM samples, whereas they presented the greatest difference in mean rank between the normal BM and AML samples.

**Figure 3:**
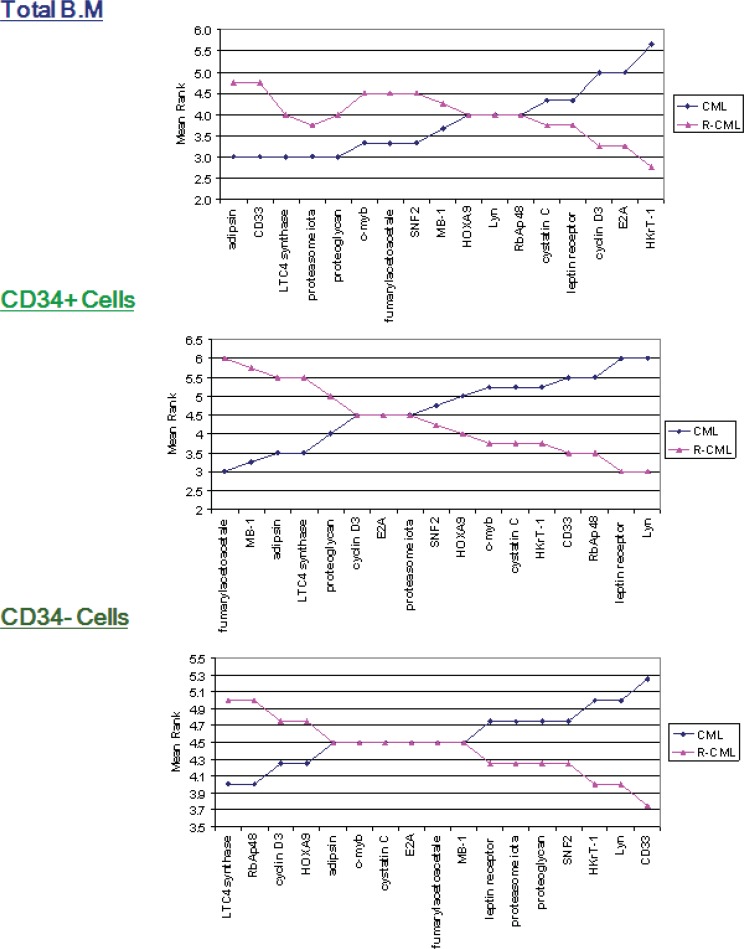
Mean ranks for Indicator genes in CML and R-CML in each of the three fractions. Mean ranks (y-axes) for each gene, calculated using the Mann–Whitney in the CML and R-CML groups (total, CD34+ and CD34-), sorted based on gene mean rank for CML

**Figure 4:**
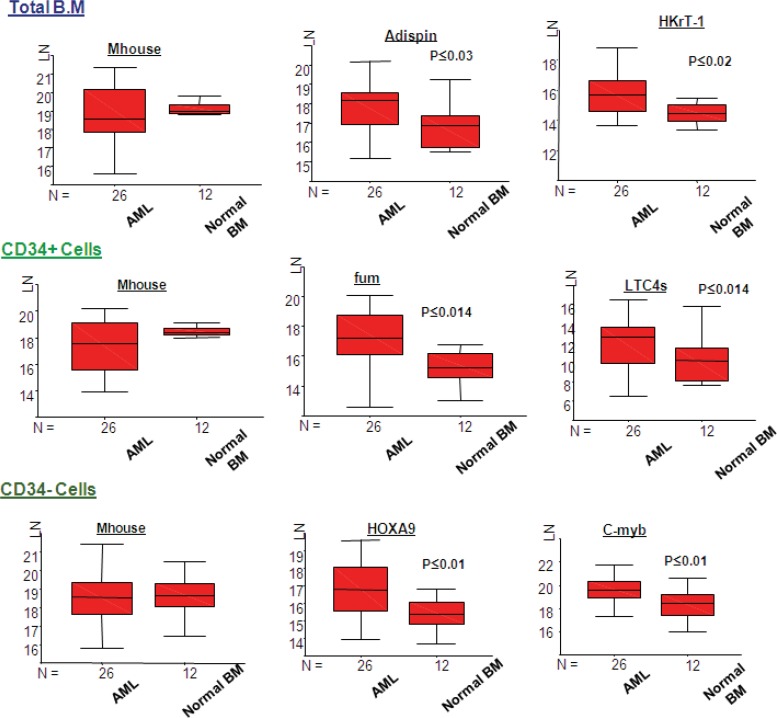
Expression levels (LN) of genes with statically significant difference between AML and Normal BM. LN, natural logarithm; N, number of samples in each group

**Figure 5:**
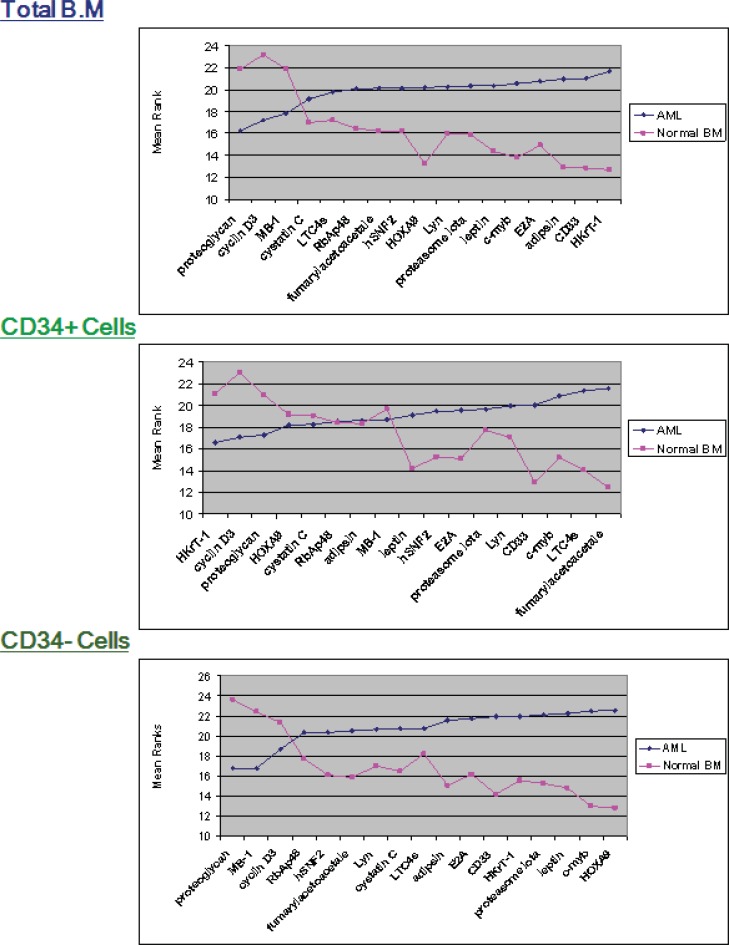
Mean ranks for Indicator genes in AML and Normal BM in each of the three fractions. Mean ranks (y-axes) for each gene, calculated using the Mann– Whitney in the AML and Normal BM groups (total, CD34+ and CD34-), sorted based on gene mean rank for AML

**Figure 6:**
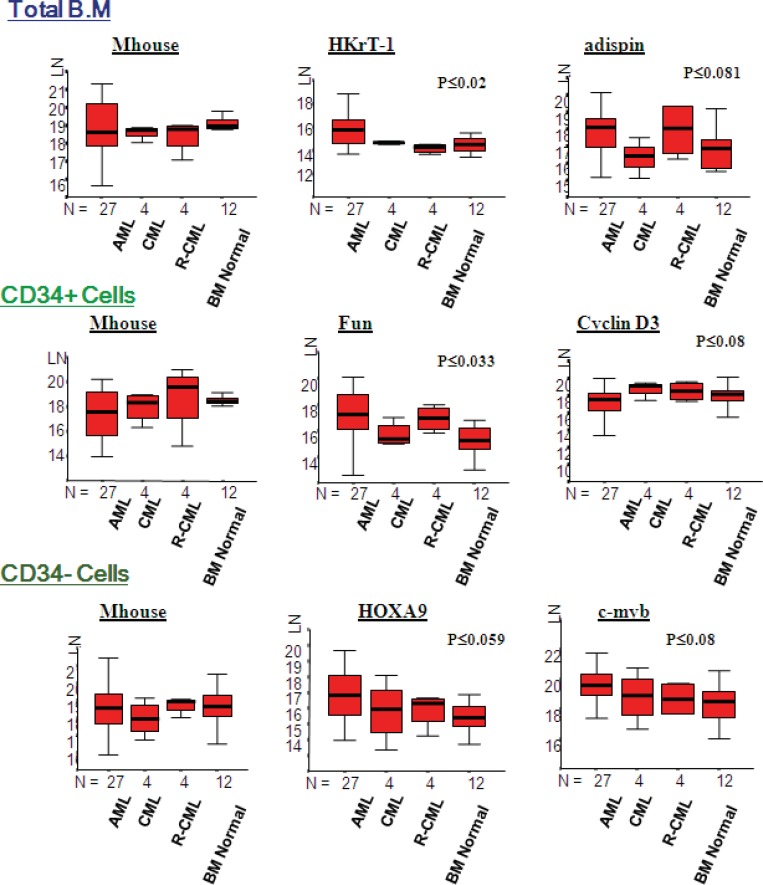
Expression levels (LN) of genes with statically significant difference between AML, CML, R-CML and Normal BM. LN, natural logarithm; N, number of samples in each group

**Figure 7:**
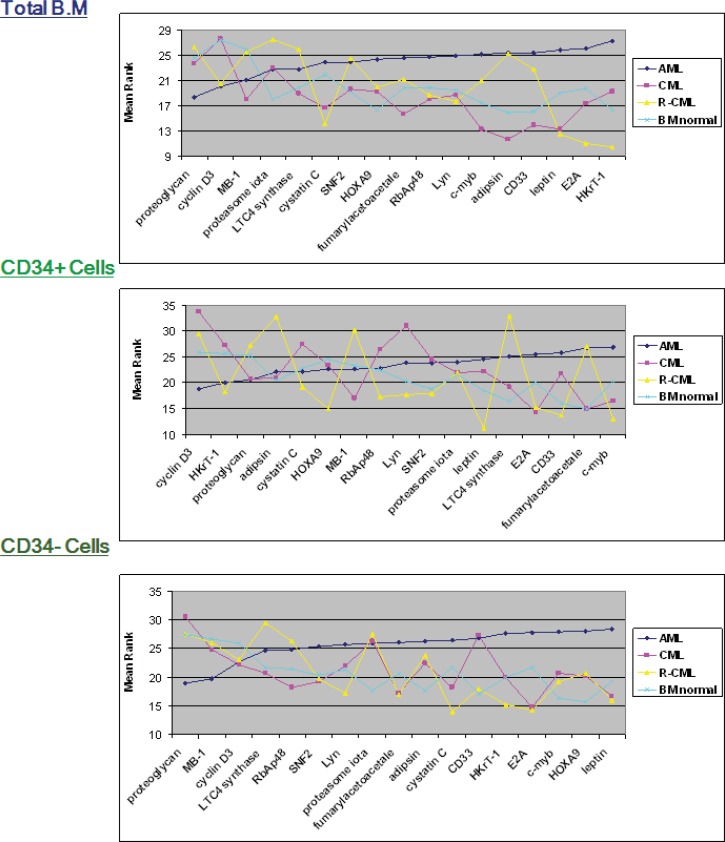
Mean ranks for Indicator genes in AML, CML, R-CML and morphologically normal bone marrow in each of the three fractions. Mean ranks (yaxes) for each gene, calculated using the Kurskal–Wallis (K–W) test in the AML, CML, R-CML and normal BM groups (total, CD34+ and CD34-), sorted based on gene mean rank for AML

**Figure 8:**
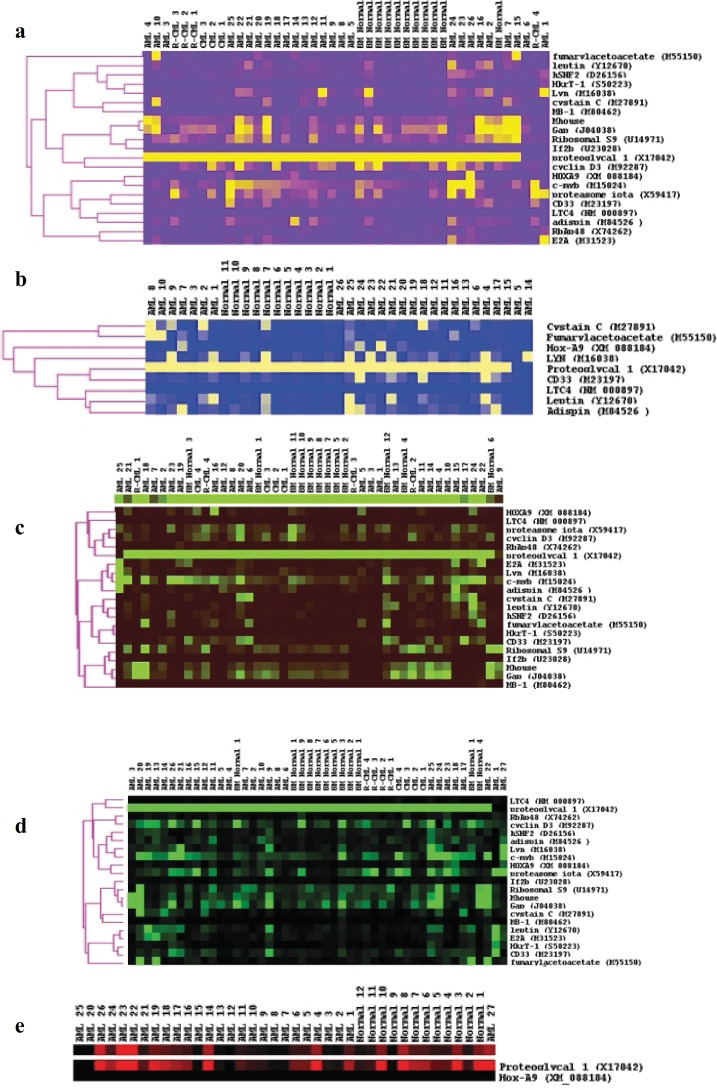
Unsupervised cluster analyses for, (a) all diagnostic groups for all genes in the total BM fraction, (b) all diagnostic groups for all genes in the CD34+ve fraction, (c) all diagnostic groups for all genes in the CD34-ve fraction, (d) AML and normal BM samples in the space of the genes upregulated in AML (from Golub *et al*, 1999), in the total BM fraction and (e) AML and normal BM samples in the space of proteoglycan 1 and HOXA9 in the CD34-ve fraction only. The normalised gene expression values for each sample were processed using the Cluster and TreeView software available from the Eisen laboratory (http://rana.lbl.gov/eisensoftware.htm).

## Discussion

In the recent years, microarray research has shown gene signatures for haematological malignancies in general, and for AML in particular (2). These findings pave the way for more specific diagnosis, prognosis, and treatment and improve patients’ survival. Therefore, in routine clinical practice a practical way for the measurement of such gene signatures is essential. Despite the fact that cDNA array methodologies are highly applicable in genes identification, expression of these genes correlates to pathology or biology. However, their application as a routine method of monitoring clinical samples is not possible, due to their high cost, fairly high amounts of starting RNA (10), and the relative low detection thresholds for individual genes.

To remove these barriers, we used a global amplification approach, known as polyA PCR (7). PolyA PCR co-ordinately amplifies cDNA copies of all polyadenylated mRNAs and creates a PCR product (polyA cDNA). Several studies have reviewed that PloyAcDNA composition shows the relative abundance of all encoded genes in the starting sample, which can be used for detection of very low amount of samples (4) involving single cells (5). The expression profiles of specific Indicator genes can be evaluated by real-time PCR by using gene specific primers and probes, and thus enabled the expression profiling of very low amounts of starting material (5). This method was assessed in BM samples from AML and ALL, and this shows the ability of the method to distinguish between these two groups using Indicator genes found in independent microarray research (6, 7). These two studies showed that, seventeen indicator genes demonstrating differential expression between AML and ALL assessed by microarray test (6) and apart from two genes that evaluated by using real time- PCR, all genes indicated similar expression level in AML and ALL to that detected in the microarray investigation (7). As a part of a previous study, samples were collected opportunistically from cases with CML and from patients with normal BM to determine the specificity of the Indicator genes measured for distinction of AML from ALL. To determine whether use of these set of Indicator genes is limited to samples of AML and ALL or they can be applied more generally in other haematological malignancies, they were also investigated in the CML and morphologically normal BM fractions..

Seventeen Indicator genes were selected from the genes discriminatory between AML and ALL found by Golub et al. (6) and the same microarray data was reassessed by Thomas et al (9). These Indicator genes were evaluated in duplicate in each sample, with 3 housekeeping genes (GAPDH, IF2-b and RbS9), and the expression extent of the 17 Indicator genes normalized to the mean of the housekeeping genes before further analysis. The mean of three housekeeping genes was utilized in order to avoid possible distortion of the findings by reliance on a single normalization gene that might experience differential expression in the different diagnostic groups. GAPDH was differentially regulated over cell cycle (11) and was transcriptional regulated in fibroblasts in early development (12) through illustration. Although it is a good Indicator of sample quality, its usage for normalization alone may introduce bias, because the diagnostic groups showed abnormal stem cell maturation.

The samples were fractionated to CD34 positive and negative, with an unfractionated total BM fraction, before RNA extraction. CD34 was demonstrated to be expressed within haematopoietic differentiation (13), specifically on primitive cells. Hence, there may be differences in gene expression in CD34 positive and negative cells (14). Identifying utilized genes from microarray analysis (6) of unfractionated BM this fraction was evaluated in the present study. For application of Indicator genes to routine diagnosis, it needs that the possible differences in gene expression between the different diagnostic groups for the different fractions are identified. However, time-consuming fractionation was avoided, if total BM showed the most significant differences between the diagnostic groups.

Despite the fact that the genes selected for the measurement were not detected in CML or normal BM, their expression was opportunistically evaluated in these two groups. Barely 1 in seventeen genes, namely cyclin D3, experienced statistically significant difference between AML and CML, in the CD34 positive fraction (Fig 1). Compared to AML, Cyclin D3 expression was seen to be significantly high in CML in the total BM samples and the CD34+ve fractions. It served a crucial role in controlling the physiological progression from the G1 to the S phase of the cell cycle and was over-expressed in several human malignancies (15), including some B-cell malignancies (16). However, it was independently shown as a signature gene for AML (17). It is interesting that in the comparison of AML and CML alone, the mean rank plots revealed least difference in the CD34 positive fraction. This demonstrates that the two conditions bear greatest similarity of gene expression in the immature fraction (Fig 2).

Although the molecular pathology of AML and CML are very different, comprising a single, uniform and well-defined translocation in CML and a variety of abnormalities in AML, the gene expression levels between the two groups failed to exhibit many significant differences. This suggests that the 17 genes chosen from the AML versus ALL analysis (6) are at least in part due to differences between myeloid and lymphoid differentiation, which may not be seen in two myeloid disorders.

However, statistical comparison amongst AML and CML and normal BM (Fig 6 and 7), and cluster analysis (Fig 8) showed distinction among AML and the others. This was demonstrated by a significant higher expression of HKrT-1 (p≤0.02) and fumarylacetoacetate (p≤0.03). These, both, were upregulated in AML in comparison with the other diagnostic groups in the total BM and CD34+ve fractions, respectively. However, the other differences were found in expression between these groups, which were nonsignificant at the level of p≤0.05. The mean rank analyses for AML, CML, and normal BM showed a similarity between the normal and CML samples, especially in the total BM fraction; however, there was a similar expression between these two groups in the CD34+ and –ve fractions (Fig 7). These findings suggested that the 17 genes were not significantly different in CML and normal BM. Some of these genes (HkrT1 and fumarylacetoacetate) experienced a significant different expression between AML and the non-AML groups, although these latter included CML. These findings highlighted the specificity of the gene signature tested, taken from an analysis of expression between AML and ALL, for distinction of AML and ALL. Thereby, these genes may be unuseful for distinction of other myeloid malignancies. This shows the specificity of microarray identified gene signatures and demonstrates the care, which must be taken in their application.

Clustering of the samples indicated predominant separation of the samples, in different fractions, into 2 clusters, one of AML samples, and a second made up of the other diagnostic groups, again confirming the similarity between the CML and normal samples, in comparison with the AML samples (Fig 8). The dominant difference between AML and the other sample types in this cohort was also shown by clustering in the space of the AML and normal samples alone, wherein all the normal samples clustered together. In addition, the distinction between AML and normal BM could also be drawn by clustering in the space of proteoglycan 1 and HOX A9 alone, in the CD34-ve fraction. HOXA9 and proteoglycan 1 also experienced the greatest difference in mean rank between the normal and AML samples, suggesting concordance of the two statistical tests.

We have previously showed the ability of the method for measurement of microarray identified gene signatures in a clinical context, by means of 17 Indicator genes selected from the work of Golub et al. (6), wherein 15 Indicator genes demonstrated similar differential expression between AML and ALL as in the original microarray investigation (7).

## Conclusion

The present study extended the measurement of the same genes to samples from cases with CML, with active disease or in remission, and to normal BM samples. The 17 genes discriminatory between AML and ALL revealed no significant differences between CML and normal samples. However, significant differences in expression were exhibited between AML and the remaining groups (CML and normal BM). This was shown by using mean rank, Mann Whitney test, and unsupervised clustering. The differences suggest specificity of the microarray gene signatures for the diagnostic distinctions wherein they were initially identified and their use outside of such a context may be uninformative. Therefore, the use of panels of microarray-identified genes should be carefully selected for different diagnostic scenarios.

## Conflict of interest

There is no conflict of interest.

## References

[1] Vardiman JW, Harris NL, Brunning RD (2002). The World Health Organization (WHO) classification of the myeloid neoplasms. Blood.

[2] Ebert BL, Golub TR (2004). Genomic approaches to hematologic malignancies. Blood.

[3] Grimwade D, Haferlach T (2004). Gene-expression profiling in acute myeloid leukemia. N Engl J Med.

[4] Brady G, Barbara M, Iscove NN (1990). Representative In vitro cDNA amplification from individual haemopoietic cells and colonies. Methods in Molecular and Cellular Biology.

[5] Al-Taher A, Bashein A, Nolan T, Hollingsworth M, Brady G (2000). Global cDNA amplification combined with real-time RT-PCR: accurate quantification of multiple human potassium channel genes at the single cell level. Yeast.

[6] Golub TR, Slonim DK, Tamayo P, Huard C, Gaasenbeek M, Mesirov JP (1999). Molecular classification of cancer: class discovery and class prediction by gene expression monitoring. Science.

[7] Sakhinia E, Faranghpour M, Liu Yin JA, Brady G, Hoyland JA, Byers RJ (2005). Routine expression profiling of microarray gene signatures in acute leukaemia by real-time PCR of human bone marrow. Br J Haematol.

[8] Núnêz C, Bashein AM, Brunet CL, Hoyland JA, Freemont AJ, Buckle AM (2000). Expression of the imprinted tumour-suppressor gene H19 is tightly regulated during normal haematopoiesis and is reduced in haematopoietic precursors of patients with the myeloproliferative disease polycythaemia vera. J Pathol.

[9] Thomas JG, Olson JM, Tapscott SJ, Zhao LP (2001). An efficient and robust statistical modelling approach to discover differentially expressed genes using genomic expression profiles. Genome Research.

[10] Glanzer JG, Eberwine JH (2004). Expression profiling of small cellular samples in cancer: less is more. Br J Cancer.

[11] Bustin SA (2000). Absolute quantification of mRNA using real-time reverse transcription polymerase chain reaction assays. J Mol Endocrinol.

[12] Moe TK, Ziliang J, Barathi A, Beuerman RW (2001). Differential expression of glyceraldehyde-3-phosphate dehydrogenase (GAPDH), beta actin and hypoxanthine phosphoribosyltransferase (HPRT) in postnatal rabbit sclera. Curr Eye Res.

[13] Bühring HJ, Asenbauer B, Katrilaka K, Hummel G, Busch FW (1989). Sequential expression of CD34 and CD33 antigens on myeloid colony-forming cells. Eur J Haematol.

[14] Chen G, Zeng W, Miyazato A, Billings E, Maciejewski JP, Kajigaya S (2004). Distinctive gene expression profiles of CD34 cells from patients with myelodysplastic syndrome characterized by specific chromosomal abnormalities. Blood.

[15] Pruneri G, Fabris S, Fasani R, Del Curto B, Capella C, Pozzi B (2003). Immunoreactivity for cyclin D3 is frequently detectable in high-grade primary gastric lymphomas in the absence of the t(6;14) chromosomal-translocation. J Pathol.

[16] Sonoki T, Harder L, Horsman DE, Karran L, Taniguchi I, Willis TG (2001). Cyclin D3 is a target gene of t(6;14) of mature B-cell malignancies. Blood.

[17] Neben K, Tews B, Wrobel G, Hahn M, Kokocinski F, Giesecke C (2004). Gene expression patterns in acute myeloid leukemia correlate with centrosome aberrations and numerical chromosome changes. Oncogene.

